# Supplementary material for: Chiral Selectivities of Permethylated α-, β-, and γ-Cyclodextrins Containing Gas Chromatographic Stationary Phases towards Ibuprofen and Its Derivatives
Source: Int J Mol Sci. 2024 Jul 16;25(14):7802. doi: 10.3390/ijms25147802 (PMC11276992; doi:10.3390/ijms25147802)
Supplement: Supplementary file 1 [file ijms-25-07802-s001.zip › ijms-3086142-supplementary.pdf]

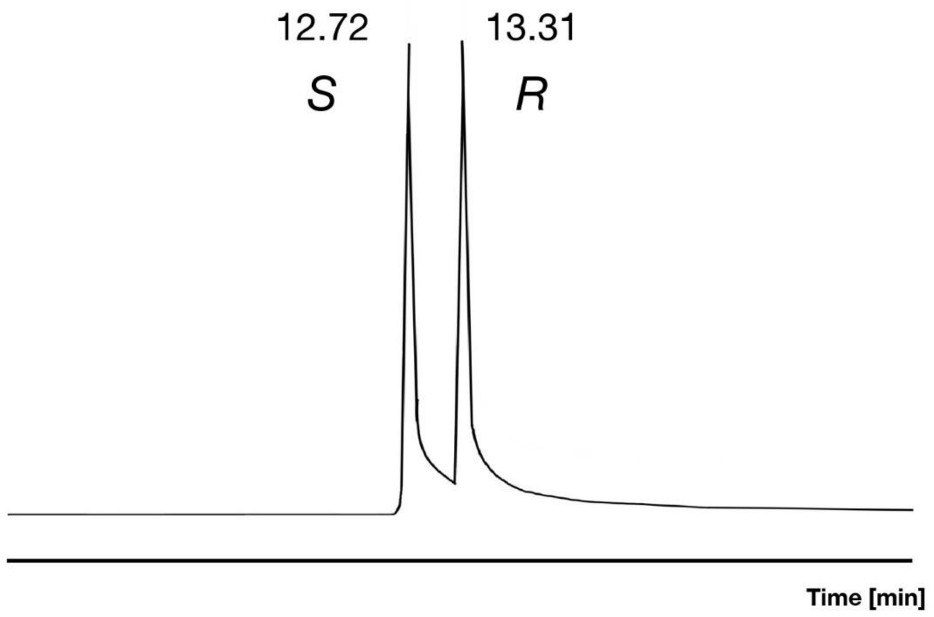

Figure S1. Chiral separations of Ibuprofen on permethylated α-CD containing stationary phase. Conditions: Instrument, Shimadzu QP5000 GC/MS; column, 25 m x 0.22 mm FSOT; stationary phase, Alpha Dex (0.25 µm); carrier, He (50 cm/sec); analysis temperature, 130° C; solvent concentration 2 mg/ml. on permethylated α -CD containing stationary phase. Enantiomeric ratio is S/R = 1.5

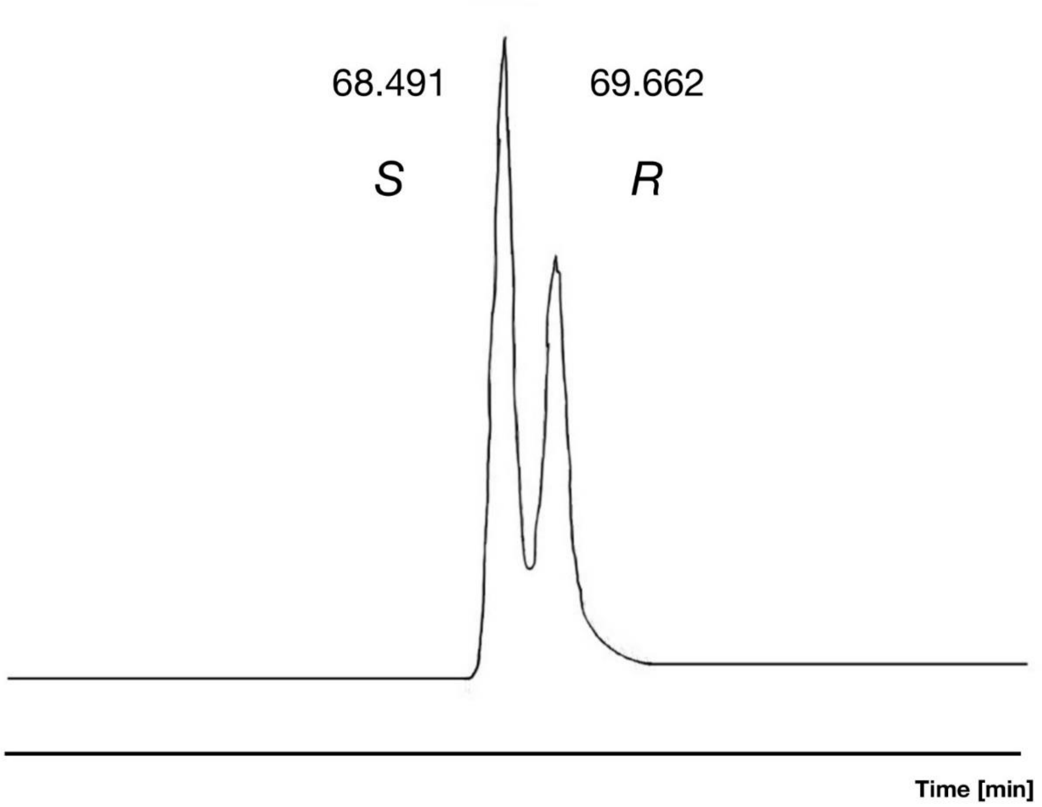

Figure S2. Partially chiral separations of Ibuprofen on permethylated γ-CD containing stationary phase. Conditions: Instrument, Shimadzu QP5000 GC/MS; column, 25 m x 0.22 mm FSOT; stationary phase, Gamma Dex (0.25 µm); carrier, He (50 cm/sec); analysis temperature, 140° C; solvent concentration 2 mg/ml. on permethylated γ-CD containing stationary phase. Enantiomeric ratio is S/R = 2.

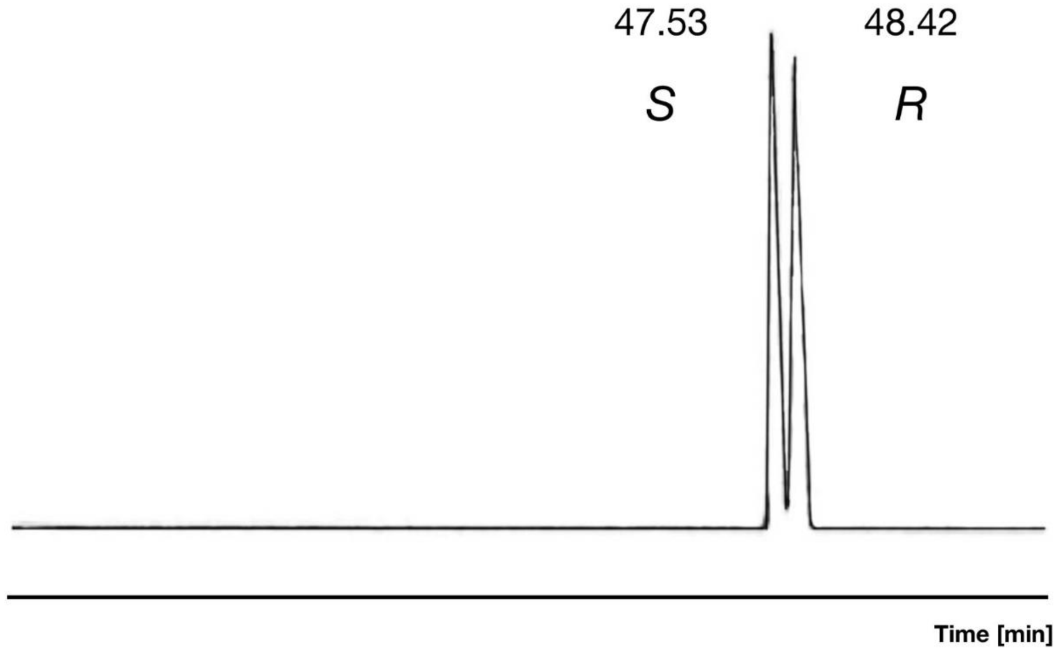

Figure S3. Chiral separations of Ibuprofen methyl ester on permethylated β-CD containing stationary phase. Conditions: Instrument, Shimadzu QP5000 GC/MS; column, 25 m x 0.22 mm FSOT; stationary phase, Cydex-B (0.25 μm); carrier, He (50 cm/sec); analysis temperature, 100° C; solvent concentration 2 mg/ml. on permethylated β -CD containing stationary phase. Enantiomeric ratio is S/R = 1 [24]

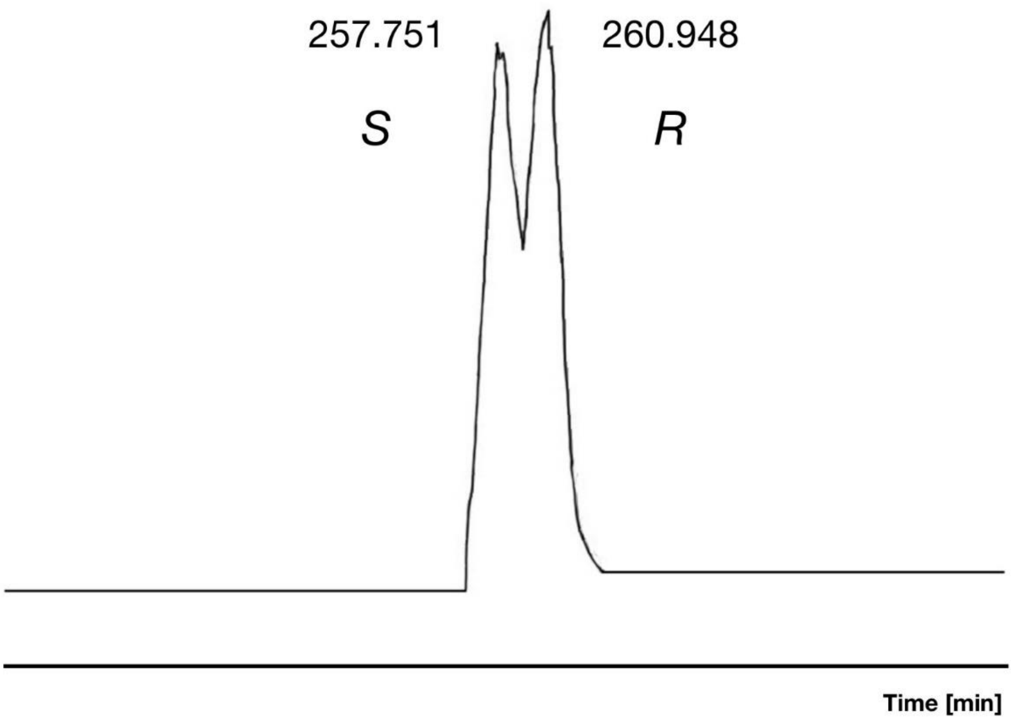

Figure S4. Partially chiral separations of Ibuprofen isobutyl ester on permethylated γ -CD containing stationary phase. Conditions: Instrument, Shimadzu QP5000 GC/MS; column, 25 m x 0.22 mm FSOT; stationary phase, Gamma Dex (0.25 µm); carrier, He (50 cm/sec); analysis temperature, 100° C; solvent concentration 2 mg/ml. on permethylated γ -CD containing stationary phase. Enantiomeric ratio is S/R = 1

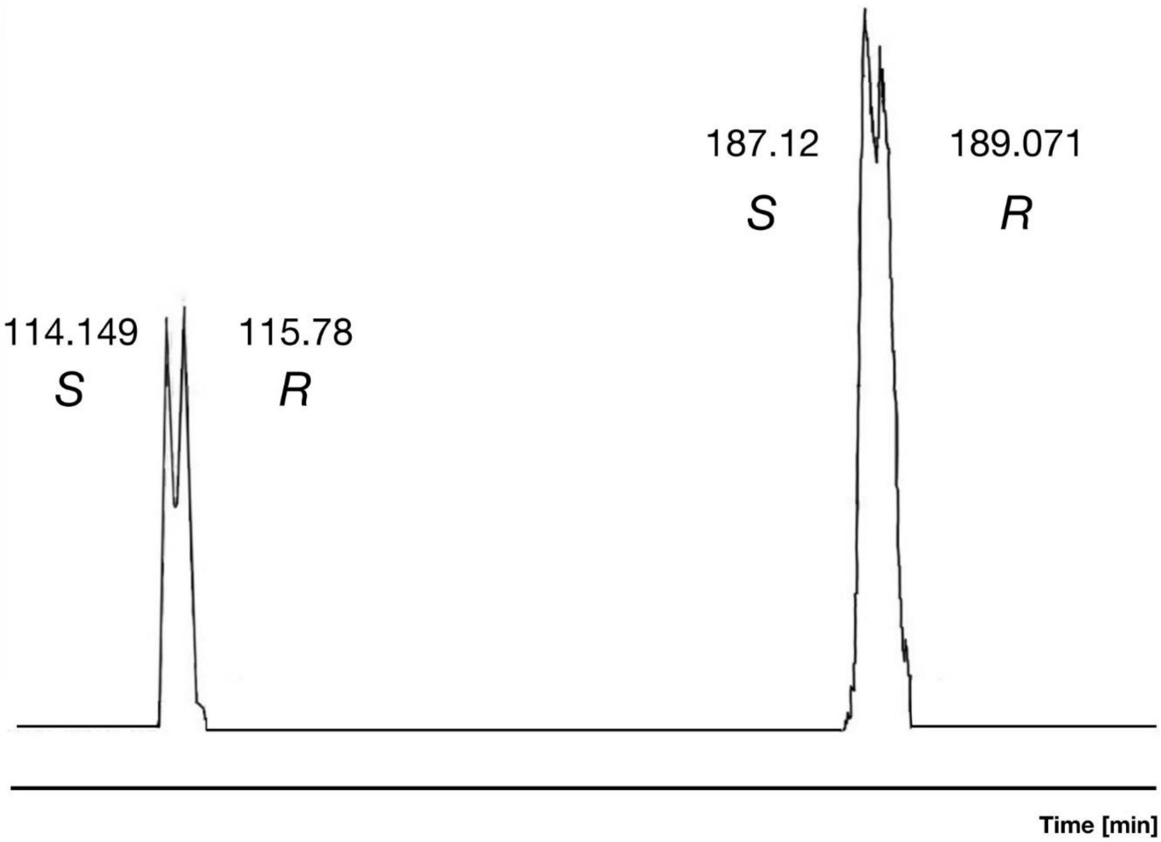

Figure S5. Chiral separations of isopropyl and propyl esters of Ibuprofen on permethylated β-CD containing stationary phase. Conditions: Instrument, Shimadzu QP5000 GC/MS; column, 25 m x 0.22 mm FSOT; stationary phase, Cydex-B (0.25 μm); carrier, He (50 cm/sec); analysis temperature, 100° C; solvent concentration 2 mg/ml. on permethylated β-CD containing stationary phase. Enantiomeric ratio is S/R = 1
